# Comprehensive Case Analysis of Tuberculous Meningitis in an Immunocompetent Patient: Diagnostic Challenges and Therapeutic Strategies

**DOI:** 10.7759/cureus.64332

**Published:** 2024-07-11

**Authors:** Rohit Sharma, Vaibhav, Diksha Yadav, Vipul Kaliraman, Aditya Duhan

**Affiliations:** 1 Department of Medicine, Pandit Bhagwat Dayal Sharma University of Health Sciences, Rohtak, Rohtak, IND; 2 Department of Neurology, Pandit Bhagwat Dayal Sharma University of Health Sciences, Rohtak, Rohtak, IND; 3 Department of Medicine, Maulana Azad Medical College, New Delhi, IND; 4 Department of Radiology, Pandit Bhagwat Dayal Sharma University of Health Sciences, Rohtak, Rohtak, IND

**Keywords:** meningeal tuberculosis, central nervous system tuberculosis, extrapulmonary tuberculosis, anti-tubercular therapy, miliary tuberculosis, tuberculous meningitis

## Abstract

Tuberculosis (TB) continues to be a significant global health concern, with India contributing substantially to the global burden. The management of TB is further complicated by HIV-associated immunodeficiency and the emergence of drug-resistant TB strains. Early diagnosis and treatment are critical, particularly for tubercular meningitis (TBM), which is among the most severe forms of extrapulmonary TB.

We present the case of a 55-year-old male who arrived at our emergency department with a one-week history of fever, headache, incoherent speech, and slurred speech. The patient had no relevant medical history or known contact with TB patients. Neurological examination revealed ptosis of the right eye and a left extensor plantar response. Laboratory investigations revealed a miliary pattern on chest radiography, and cerebrospinal fluid analysis showed an adenosine deaminase (ADA) level of 14.4 U/L, a total cell count of 110/mm³, glucose of 6 mg/dL, and protein of 228.4 mg/dL, supporting the diagnosis of TBM. Magnetic resonance imaging (MRI) indicated brain lesions consistent with TBM.

TBM represents the most devastating form of extrapulmonary TB if left untreated. Therefore, prompt initiation of antitubercular therapy and continued vigilance in endemic regions are essential for addressing this complex global health issue.

## Introduction

Tuberculosis (TB) represents a significant and pervasive global public health challenge. According to the World Health Organization's 2021 report, eight countries are responsible for over two-thirds of the global TB burden, with India contributing a staggering 28% of all reported cases [1]. 

Although tuberculous involvement of the central nervous system (CNS) is relatively infrequent, it imposes a significant burden, resulting in severe morbidity among affected individuals. This issue is exacerbated by the high prevalence of immunodeficiency linked to the human immunodeficiency virus (HIV) pandemic, which creates an environment conducive to the dissemination of TB. Furthermore, the challenge is compounded by the high prevalence of multidrug-resistant TB, impacting approximately 8.26 per 100,000 population [2].

Given the intricate interplay of these factors, the urgency of early TB diagnosis and treatment cannot be overstated. Proactive measures are crucial to minimize the potential for complications and sequelae associated with this global health dilemma. Delays in recognizing and treating tuberculous meningitis (TBM) can result in severe disabilities and poor outcomes. Therefore, it is imperative to acknowledge and address this presentation of the disease, especially in countries where TBM is highly endemic.

## Case presentation

A 55-year-old male patient, a shopkeeper by occupation, was admitted to our ward with a one-week history of low-grade fever, headache, incoherent speech, and slurred speech. He had no significant medical history or prior treatment history. There was no history of TB contact. The patient was a non-smoker, non-alcoholic, and had no history of drug abuse.

Upon admission, the patient was disoriented, with a Glasgow Coma Scale (GCS) score of 13 (E4V4M5), and had a recorded fever of 101°F. No rash, petechiae, or eschar was observed. The respiratory, cardiovascular, and abdominal examinations were within normal limits. Neurological examination revealed ptosis of the right eye and a left extensor plantar response. Further examination showed normal power, tone, and reflexes in the upper and lower limbs. Bilateral pupils were normal in size and reactive to light. There were no cerebellar signs or indications of other cranial nerve involvement.

**Figure 1:**
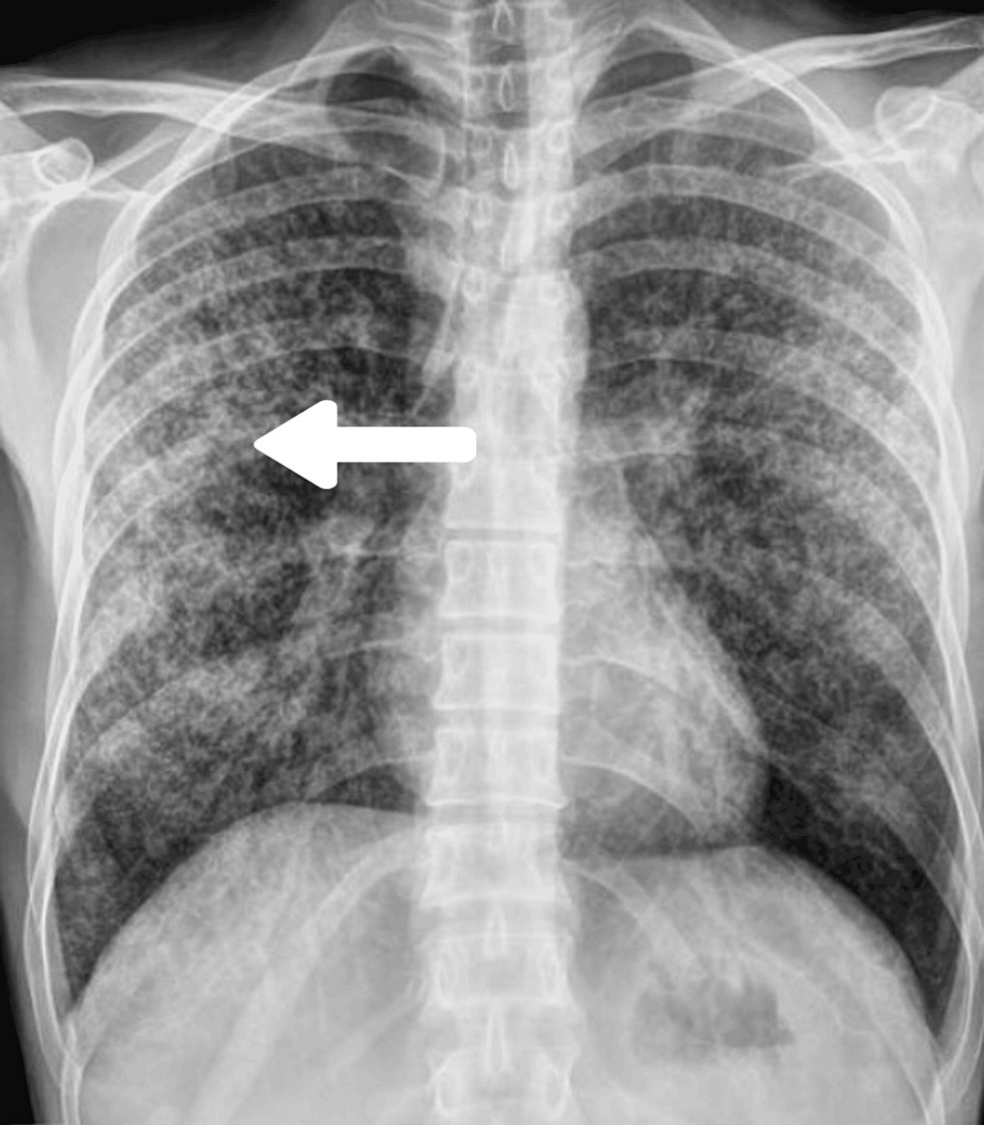
Chest radiograph showing miliary pattern (white arrow) which is suggestive of tuberculosis

Laboratory investigations revealed a hemoglobin level of 11.4 g/dL, a total leukocyte count of 12,400/mm³ with 86% neutrophils, 6% lymphocytes, and 8% monocytes, and a platelet count of 160,000/µL. Fasting blood sugar was 130 mg/dL. Blood pressure was 100/70 mmHg, pulse rate was 60/min, and oxygen saturation at room air was 97%. Blood gas analysis showed a sodium level of 127.2 mmol/L and a potassium level of 3.35 mmol/L. Rapid kit tests for HIV, Hepatitis B surface antigen (HBsAg), and Hepatitis C virus (HCV) were negative. A chest radiograph displayed a miliary pattern suggestive of disseminated TB (Figure 1).

Lumbar puncture results were consistent with TBM, showing an adenosine deaminase (ADA) level of 14.4 U/L (reference range: <5 U/L), total cell count of 110/mm³, glucose level of 6 mg/dL, and protein level of 228.4 mg/dL (reference range: 15-40 mg/dL). Further investigations, including contrast-enhanced magnetic resonance imaging (CEMRI) of the head, revealed multiple small nodular lesions scattered throughout the brain suggestive of tuberculomas and a subacute infarct in the right fronto-temporo-parietal lobe. Following the confirmation of TBM, the patient was initiated on anti-tubercular therapy (Rifampicin 600 mg, Isoniazid 300 mg, Ethambutol 1200 mg, and Pyrazinamide 1600 mg) and corticosteroids (Dexamethasone 4 mg intravenously every six hours).

## Discussion

We report a case of TBM in an immunocompetent patient who was previously healthy. A noteworthy 17% of TB cases manifest as extrapulmonary in nature. Common sites of extrapulmonary TB include lymph nodes, pleura, genitourinary tract, bones and joints, meninges, peritoneum, and pericardium. TBM constitutes about 1% of all TB cases in India, which equates to approximately 17,000 cases per year [1].

In the majority of cases, the spread of infection in TBM is believed to occur via the hematogenous route. This pathological condition is characterized by the accumulation of gelatinous exudates within the meninges, often leading to the involvement of cranial nerves. The exudate is composed of various cellular components, including mononuclear cells, epithelioid cells, and Langhans' giant cells [3].

Ependyma is the epithelial lining of the brain's ventricles and central canal of the spinal cord. TBM typically begins in subependymal regions, areas adjacent to ependyma, originating from small, cheese-like lesions called "Rich" foci. The accumulation of copious exudates in TBM can lead to obstruction in Cerebrospinal Fluid (CSF) flow, particularly at the level of the basal cisterns. Basal cisterns are CSF-filled spaces located at the base of the brain, particularly around the brainstem and adjacent to the cerebral hemispheres. This condition, termed “basal arachnoiditis,” can result in the development of communicating hydrocephalus, which is a condition characterized by excessive accumulation of CSF within the brain's ventricular system, which can lead to enlargement of the ventricles and potentially increased intracranial pressure [4].

A high incidence of hydrocephalus, up to 80%, has been reported in cases of TBM [5]. This complication underscores the severity and clinical significance of TBM and highlights the importance of timely diagnosis and intervention. In our patient, hydrocephalus was not present, so there was no need for a ventriculo-peritoneal shunt. Ventriculo-peritoneal shunts are devices used in the treatment of hydrocephalus by shunting CSF from ventricles. A catheter is surgically placed into the ventricle, with an external segment connected to a pressure-regulating valve. The distal catheter extends under the skin, directing CSF into the peritoneal cavity where it is reabsorbed.

Various diagnostic algorithms have been published for the clinical diagnosis of TBM. For example, the classification of TBM can be based on the severity of the disease [6].

Stage 1: The patient is fully conscious.

Stage 2: The patient is drowsy or has focal neurological signs.

Stage 3: The patient is comatose or nearly comatose.

The diagnosis of TBM represents a significant clinical challenge. It is imperative to maintain a vigilant clinical approach to promptly initiate appropriate anti-tubercular therapy. The definitive diagnosis of TBM hinges upon the identification of tubercle bacilli within the CSF. This identification can be achieved through smear examination or bacterial culture, particularly in individuals exhibiting clinical symptoms or signs suggestive of the disease. CSF analysis typically shows elevated protein levels, predominantly lymphocytes, and reduced glucose levels. In most cases, CSF protein levels range between 100 and 500 mg/dL. It is important to perform at least three lumbar punctures, extracting about 10-15 mL each time, with one performed daily. The challenge in diagnosing TB lies in the fact that it takes six to eight weeks for *Mycobacterium bacilli* to appear in culture. Elevated ADA levels are often found in the body fluids, including CSF, of TB patients [7,8].

The GeneXpert test, recommended by the World Health Organization, uses real-time polymerase chain reaction (RT-PCR) to detect *Mycobacterium tuberculosis*. It should be quickly utilized in CSF or sputum samples of suspected TB patients. In CSF samples, the test has demonstrated a sensitivity of 79% and a high specificity of 98% [9]. For sputum samples, it has shown a sensitivity of 81% and a high specificity of 98%, as demonstrated in early studies on patients with pulmonary TB [10]. Moreover, this test not only rapidly detects *M. tuberculosis* but also identifies rifampicin resistance, providing crucial information for timely and effective treatment [11].

TBM is the most devastating form of extrapulmonary TB if left untreated. Even with standard anti-tuberculous treatment, the short-term mortality rate ranges from 20% to 69% [12]. Early diagnosis and treatment can significantly reduce the high mortality associated with this disease.

## Conclusions

TBM remains a critical and severe manifestation of extrapulmonary TB, necessitating prompt diagnosis and intervention. This case of a 55-year-old immunocompetent male underscores the importance of comprehensive diagnostic approaches, including clinical evaluation, chest radiography, CSF analysis, and MRI. The identification of miliary patterns on radiography, elevated ADA levels, and characteristic CSF findings were pivotal in diagnosing TBM. Despite the absence of hydrocephalus, the patient's management with anti-tubercular therapy and corticosteroids highlights the need for timely and targeted treatment to mitigate the high morbidity and mortality associated with TBM. Continuous vigilance and proactive measures are essential in regions endemic to TB to improve patient outcomes and address this significant global health challenge.
